# Cephalic Index in Indigenous Tharu Community

**DOI:** 10.31729/jnma.3487

**Published:** 2018-10-31

**Authors:** Sanzida Khatun

**Affiliations:** 1 Department of Anatomy, Nobel Medical College, Biratnagar, Nepal

**Keywords:** *cephalic index*, *head breadth*, *head length*, *head shape*, *head type*

## Abstract

**Introduction:**

Cephalic index is an important parameter for differentiation of race and sex which varies significantly on the basis of hereditary, geographical, racial, sexual and other factors. It is a morphometric expression of different forms of head. The objective of this research was to evaluate the cephalic index of people of indigenous Tharu community of Biratnagar, Nepal and to determine different head types.

**Methods:**

A cross-sectional study was conducted in which maximum head length and breadth of 100 adult Tharu people living in Biratnagar were measured using spreading caliper and scale. The cephalic index was obtained from these values using Hrdlicka's method.

**Results:**

The mean cephalic index of the study population was 75.99±4.97. The mean cephalic indices of males and females were 76.22+5.14 and 75.78±4.85 respectively. The most common head type observed was dolichocephalic type 47 (47%). It was followed by mesocephalic type 37 (37%), brachycephalic type 13 (13%) was less common and least common was hyperbrachycephalic type 3 (3%).

**Conclusions:**

Long head (dolichocephalic) type is more common in Tharu population in both the genders, whereas, broad head (brachycephalic and hyperbrachycephalic) type is present in very few people.

## INTRODUCTION

Cephalometry is the science in which dimensions of head and face are measured. It is a branch of anthropometry.^1^ Anthropometry refers to morphological trait of human being which can be measured externally.^2^ Cephalometry has deep roots in criminology and enables diagnostic comparison between patients and normal population.^1^

Cephalic index (CI) is an important parameter for differentiation of race and sex and varies with heredity and geography.^1^ On its basis, the head shapes can be classified into dolichocephalic, mesocephalic, brachycephalic and hyperbrachycephalic types.^3^ The study population in this study are people of an ethnic group, Tharu people with typical morphological traits, living in southern foothills of Himalayas and are indigenous to Terai region of Nepal.^4, 5^

The present study was aimed at evaluating the cephalic index in people of Tharu community of Biratnagar, Nepal and determining different head types and gender differences.

## METHODS

A descriptive cross-sectional study was conducted in Tharu community living in Biratnagar, Morang district of Nepal from June 2017 to March 2018. The ethical approval (ref. no. 65/2017) was taken on 12^th^ June, 2017 from Institutional Research Committee of Nobel Medical College, Biratnagar, Nepal. The subjects included adults of age 18 and above years. Selection of adults as study group was due to the fact that the morphology of human body is not stable during earlier years of life and gets stable overtime only during adulthood. Individuals with any acquired or congenital cranio-facial deformities were excluded from the study. A written consent was obtained from all the subjects after providing them information regarding procedure and the benefits of the study.

Sample size was calculated using following formula for estimating population mean for cross-sectional study:


$$\text{n =}{\text{z}}^{2}\times \sigma^{2}/{\text{d}}^{2}$$

where, n is the required number of sample size z is the factor to achieve 95% level of confidence is the predicted value of the population standard deviation of cephalic index, (*a* =5.035 taken from previous studies^1^) d is the absolute margin of error (d=2)

The sample size derived was 24.34. However, the study was done in 100 individuals to improve validity of the results.

The method chosen for assessing cephalic index was Hrdlicka's method as this method has been used by a number of scientists in their studies.^6^ The materials used in the study were: a spreading caliper, a measuring scale and a pencil. The subjects were allowed to sit on a chair in a relaxed state with head in anatomical position. The following measurements were taken between the specified points with the help of the spreading caliper. The measurements taken were in centimeters.

Head length (HL) (maximum anteroposterior diameter) = Glabella to inion

Head breadth (HB) (maximum transverse diameter) = Distance between maximum elevations at two parietal eminences

On the basis of international description (William et al),^3^ the formula used for calculating the cephalic index was:


Cephalic Index = (Head Breadth/Head Length)×100

On the basis of cephalic index (CI), head types were classified as dolichocephalic (CI<75), mesocephalic (75<CI<80), brachycephalic (80<CI<85) and hyperbrachycephalic (CI>85) types.

The data were computed and analyzed with the help of statistical package for social sciences (SPSS) 16 software. The descriptive analysis of the data was done to derive minimum, maximum, mean and standard deviation of head length, head breadth and cephalic index of both sexes. Student's t-test was performed to compare the means of the variables between males and females.

## RESULTS

In present study, there were 100 individuals with age of 18 years and above consisting of 45 (45%) male and 55 (55%) female.

According to the data obtained from the study, the head length of the subjects ranged from 15.00 cm to 20.00 cm with mean of 17.70±1.03 cm. The head breadth ranged from 11.30 cm to 16.00 cm with mean of 13.43±0.89 cm. The mean cephalic index was 75.99±4.97.

Table 1enlists all statistics from the research showing head length, head breadth and CI. Among them, the mean head length was 18.38±0.82 cm in males, while in females, it was 17.14±0.82cm. Similarly, the mean head breadth was 13.98±0.81cm in males, while in females, it was 12.97 (±0.66) cm. The mean cephalic index in males was 76.22±5.14, while in females, it was 75.79±4.85. Although the mean head length (P<0.001) and mean head breadth (P<0.001) between males and females were found to be statistically significant, cephalic index between males and females was found to be statistically insignificant (P = 0.66)

**Table 1:** Statistics showing head length, breadth and cephalic index.

		Mean±SD	Minimum	Maximum	
Head Length (cm)	Male	18.38±0.82	16.00	20.00	<0.001
	Female	17.14±0.82	15.00	18.80	
	Total Population	17.70±1.03	15.00	20.00	
	Male	13.98±0.81	12.30	16.00	
Head Breadth (cm)	Female	12.96±0.66	11.30	14.60	<0.001
	Total population	13.43±0.89	11.30	16.00	
Cephalic Index	Male	76.22±5.14	63.40	89.38	0.66
	Female	75.79±4.85	66.47	88.34	
	Total population	75.99±4.97	63.40	89.38	

**Figure 1.**
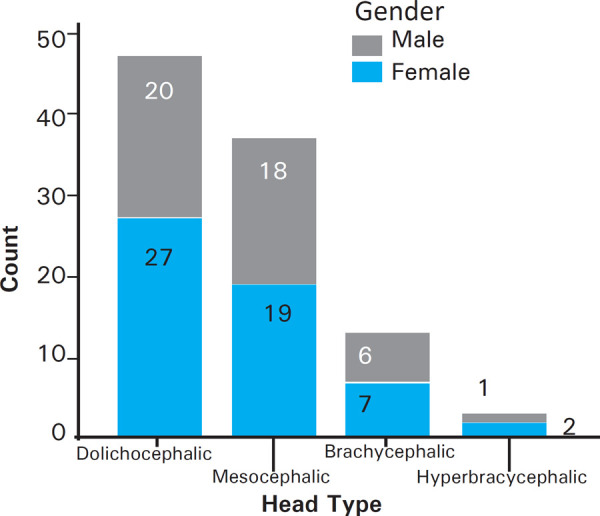
Bar-diagram showing distribution of head types.

The head type evaluated on the basis of cephalic index showed that the most common type was dolichocephalic type 47 (47.0%) followed by mesocephalic 37 (37.0%), brachycephalic 13 (13.0%) and the least common was hyperbrachycephalic 3 (3.0%) types (Figure 1).

In both sexes, the pattern was similar. The most common head type in males was dolichocephalic 20 (44.4%) followed by mesocephalic 18 (40.0%), brachycephalic 6 (13.3%) and the least common was hyperbrachycephalic 1 (2.2%). Similarly, the most common head type in females was dolichocephalic 27 (49.1%) followed by mesocephalic 19 (34.5%), brachycephalic 7 (12.7%) and the least common was hyperbrachycephalic 2 (3.6%) as shown (Table 2).

**Table 2.** Frequency of head types in both sexes.

	Male n (%)	Female n (%)	Total population n (%)
Dolichocephalic	20 (44.4%)	27 (49.1%)	47 (47.0%)
Mesocephalic	18 (40.0%)	19 (34.5%)	37 (37.0%)
Brachycephalic	6 (13.3%)	7 (12.7%)	13 (13.0%)
Hyperbrac-hycephalic	1 (2.2%)	2 (3.6%)	3 (3.0%)
Total	45 (100.0%)	55 (100.0%)	100 (100.0%)

## DISCUSSION

The present study reports the evaluation of cephalic index and head types in the people of Tharu community living in Biratnagar, Nepal. It was observed in various literature that a number of concepts have been followed in studies related to cephalic index. Some researchers were interested in comparing some anthropological factors with intelligence^7^ whereas some were comparing the data of newer generations with older generations^8^ and some were comparing the indices in different groups and ethnicity in same country.^9^ However, in this study, it has been attempted to study the cephalic indices and head types of a select community only, the indigenous Tharu people living in Biratnagar, Nepal.

The mean head length and head breadth of male observed in this study were 18.38±0.82 cm and 13.99±0.82 cm respectively. The head length value is very close to the findings revealed in the study performed in Gujarati (18.26 cm)^9^ and Gurung (18.00 cm)^11^ communities but the head breadth was found to be significantly lower than theirs. The mean head length and breadth, however, is found to be lower than female Gurungs.^9^ The mean head length and breadth of both sexes is significantly lower than that of Croatian medical students.^8^

The mean cephalic index observed in this study was 75.99±4.97. This finding is lower than the findings observed in many other studies which reveal mean cephalic indices of 80.81, 83.70 and 79.38.^9, 11, 13^ In contrast to these reports, the findings of Tomljanovi AV^8^ and that of Eroje MA^12^ reveal lower cephalic indices. The mean cephalic index among males, in this study, is found to be 76.22±5.14 which is close to few other studies performed in India.^10, 13^ However, in many other studies, the mean cephalic index among males is found to be higher than that in this study.^9, 11^ In contrast to these findings, the finding of Ogbia tribe shows lesser mean cephalic index of 73.68.^12^ The mean cephalic index among females, in the present study, is 75.79 which is higher than that of Ogbian tribe's females and lower than findings in other studies performed in population of Nepal and India.^9, 11, 13^ In this study, cephalic index between males and females was found to be statistically insignificant (P = 0.66). In contrast to this finding, other studies performed in Nepal revealed statistically significant value of cephalic index between male and female.^11, 14^

In this study, it was observed that dolichocephaly was the most common head type. This finding is consistent with findings of other studies performed in Croatia and Nigeria.^8, 12^ On the contrary, brachycephaly was reported as most common head type in other studies conducted in Nepal and in another study conducted in India, brachycephaly and dolichocephaly were equally frequent (33%) followed by mesocephaly (27%).^11, 13, 14^ On the other hand, in other studies conducted in parts of India, most common head type reported was mesocephalic type.^9, 10^

Many researchers have raised the topic of “brachycephalisation” in their studies.^8, 9, 11, 13^ It is the tendency of the brain to grow more towards the lateral direction.^13^ This kind of change in dimension and form of head could be influenced by environmental factors such as climate, nutrition and position of body.^15^ Additional research will be required to determine such tendencies of human body to change in such a trend and continuous effort shall be done to address such facts in our future studies.

## CONCLUSIONS

Dolichocephalic head type is most prevalent in Tharu community whereas brachycephalic type is less common and hyperbrachycephalic type is rare.
